# Incidence of *Brucella* infection in various livestock species raised under the pastoral production system in Isiolo County, Kenya

**DOI:** 10.1186/s12917-021-03036-z

**Published:** 2021-10-30

**Authors:** Josiah Njeru, Daniel Nthiwa, James Akoko, Harry Oyas, Bernard Bett

**Affiliations:** 1 International Livestock Research Institute, Nairobi, Kenya; 2 Department of Biological Sciences, University of Embu, Embu, Kenya; 3 Department of Biomedical Sciences and Technology, Maseno University, Kisumu, Kenya; 4 Veterinary Epidemiology and Economics Unit, Directorate of Veterinary Services, Ministry of Agriculture, Livestock and Fisheries, Nairobi, Kenya

**Keywords:** *Brucella* spp., Incidence, Livestock species, Kenya

## Abstract

**Background:**

We implemented a longitudinal study to determine the incidence of *Brucella* infection in cattle, camels, sheep and goats that were being raised in a pastoral area in Isiolo County, Kenya. An initial cross-sectional survey was implemented to identify unexposed animals for follow up; that survey used 141 camels, 216 cattle, 208 sheep and 161 goats. Sera from these animals were screened for *Brucella* spp. using the Rose Bengal Plate test (RBPT), a modified RBPT, and an indirect multispecies Enzyme Linked Immunosorbent Assay (iELISA). Results of RBPT and iELISA were interpreted in parallel to determine seroprevalence. A total of 30 camels, 31 cattle, 22 sheep and 32 goats that were seronegative by all the above tests were recruited in a subsequent longitudinal study for follow up. These animals were followed for 12 months and tested for anti-*Brucella* antibodies using iELISA. Seroconversion among these animals was defined by a positive iELISA test following a negative iELISA result in the previous sampling period. All seropositive samples were further tested using real-time PCR-based assays to identify *Brucella* species. These analyses targeted the *alkB* and *BMEI1162* genes for *B. abortus*, and *B. melitensis,* respectively. Data from the longitudinal study were analysed using Cox proportional hazards model that accounted for within-herds clustering of *Brucella* infections.

**Results:**

The overall incidence rate of *Brucella* infection was 0.024 (95% confidence interval [CI]: 0.014–0.037) cases per animal-months at risk. *Brucella* infection incidence in camels, cattle, goats and sheep were 0.053 (0.022–0.104), 0.028 (0.010–0.061), 0.013 (0.003–0.036) and 0.006 (0.0002–0.034) cases per animal-month at risk, respectively. The incidence rate of *Brucella* infection among females and males were 0.020 (0.009–0.036) and 0.016 (0.004–0.091), respectively. Real-time PCR analyses showed that *B. abortus* was more prevalent than *B. melitensis* in the area*.* Results of multivariable Cox regression analysis identified species (camels and cattle) as an important predictor of *Brucella* spp. exposure in animals.

**Conclusions:**

This study estimated an overall brucellosis incidence of 0.024 cases per animal-months at risk with camels and cattle having higher incidence than sheep and goats. These results will inform surveillance studies in the area.

**Supplementary Information:**

The online version contains supplementary material available at 10.1186/s12917-021-03036-z.

## Background

Brucellosis is an important zoonotic disease that affects a great variety of hosts such as livestock (cattle, sheep, goats and camels), humans and wildlife [1]. Whereas this disease has been successfully controlled or eradicated in livestock populations in many developed countries including New Zealand, Japan and Australia [2], it remains a major problem affecting both livestock production and humans in Kenya [3], and also other parts of Africa [4]. Brucellosis causes direct production losses resulting from abortions, stillbirths, infertility, the mortality of calves/kids/lambs, longer calving intervals, reduced draught power, poor weight gain, and reduced milk production [5]. The etiological agent of this disease is an intracellular gram-negative coccobacillus of the genus *Brucella*. The main *Brucella* spp. that affect livestock species include *B. abortus* (cattle, camels), *B. melitensis* (sheep, goats)*, B. suis* (pigs), and *B. ovis* (sheep) [1]. Humans serve as incidental hosts for *Brucella* spp. with *B. melitensis*, *B. abortus*, *B. suis* and *B. canis* being the main pathogenic species [6]. While *Brucella* spp. may show host preference, inter-species transmission of this pathogen may occur through spill-over in areas with intense interactions between livestock and wildlife [7], or in mixed livestock production systems [8]. For example, cattle are often infected by *B. suis* and *B. melitensis* [9]. *B. abortus* has also been detected in pigs [10] and small ruminants (sheep and goats) [11].

There are limited studies that have been carried out to understand the epidemiology of *Brucella* spp. in Kenya [3] even though this pathogen is known to be endemic in pastoral areas [12]. Many seroprevalence studies have been done in the country involving livestock and humans. In pastoral areas, seroprevalences in humans often range between < 1–46.5% [3], while in livestock, they range between 3 and 40% [3, 8, 13, 14]. Seroprevalance in livestock is often associated with advanced age, large herd sizes, communal herding, and pastoralism [8, 12, 14], while in humans, advanced age, consumption of raw meat or unpasteurised milk and poor access to health services are known risk factors [2, 15]. Although seroprevalence estimates provide useful insights on the distribution of burden, they may be confusing for some diseases like brucellosis whose antibodies persist in circulation for months following recovery of the infection. In such cases, measures of incidence would provide more realistic indicators of burden.

There are also major challenges with screening of *Brucella* in humans and animals. This is a major limitation in remote areas due to limited veterinary and animal health personnel, poor laboratory infrastructure, and lack of biocontainment facilities required for culturing the agent [16, 17]. Due to these limitations, the diagnosis of brucellosis in animals is mainly performed using serological tests such as Rose Bengal Plate Test (RBPT), Milk Ring Test (MRT), Serum Agglutination Test (SAT), Complement Fixation Test (CFT), Enzyme Linked Immunosorbent Assay (iELISA), Competitive Enzyme Immunoassays and Fluorescence Polarization Assay (FPA) which can be performed in laboratories with simple equipment [17]. Among these tests, RBPT is more convenient for low-income countries since it is less technologically demanding, less expensive and yields good results if standardized correctly under local conditions with proven bacteriological samples [18]. These serological tests are often used in series or parallel depending on the overall objective of the study. Whereas the parallel testing of sera increases the overall diagnostic sensitivity, this testing strategy also reduces specificity unlike in series testing.

This study was implemented to determine the seroprevalence and incidence of *Brucella* infection in cattle, camels, sheep, and goats raised in a common (pastoral) area in Isiolo County, northern Kenya. Our study further identified the potential risk factors associated with *Brucella* spp. seroprevalence and incidence in animals as well as the main *Brucella* species circulating among livestock animals in the area. Our findings form the basis for further One Health surveillance studies.

## Results

### Descriptive results

A total of 841 animals consisting 382 cattle, 185 sheep, 174 goats and 100 camels were sampled in the cross-sectional survey. The total number of cattle and camel herds sampled were 10 and 3 respectively, while sheep and goat flocks were 8 and 10, respectively. The overall median herd size for all animals was 25 (range; 8–110) while those of cattle and camels were 26 (range; 8–110) and 40 (range; 10–50), respectively. For sheep and goats, the median flock sizes were respectively 17.5 (range; 2–51) and 11 (range; 3–64). A considerable proportion of the sampled animals, 309 (36.7%) were not included in the analysis due to either missing epidemiological data or they were not tested using RBPT and mRBPT due to logistical constraints. The overall apparent seroprevalence of *Brucella* spp. at animal-level was 11.3% (95% CI; 8.6–14.0, *n* = 532) based on the parallel interpreted results of the conventional RBPT and iELISA tests. In decreasing order, the true seroprevalences of *Brucella* spp. in camels, cattle, goats and sheep were 23.7% (95% CI; 3.36–50), 14.4% (95% CI; 10.3–18.1), 13.5% (95% CI; 8.2–21.4) and 2.7% (95% CI; 0.9–7.8), respectively. The apparent seroprevalences of *Brucella* spp. by the animal-level independent factors used in the study are presented in Table 1. These varied significantly between the livestock species (Fisher’s exact 2-tailed *P* = 0.002) and sex (*P* = 0.027). More female animals (12.6%; 95% CI; 9.7–16.0) tested positive for *Brucella* spp. antibodies compared to males (4.5% 95% CI 1.1–8.4) (Table 1). We also found a statistically significant difference between *Brucella* spp. seroprevalence and the age of animals (*P* = 0.002); adult animals had higher seroprevalence (13.4%; 95% CI; 10.3–17.0factors associated with the seroprevalance of Brucella spp. based on the univariable mixed) compared to weaners (6.7%; 95% CI; 1.8–16.2) and young animals (0.0%) (Table 1). Results on cross-tabulation of *Brucella* spp. seroprevalence by species, sex, age and pregnancy status are summarized in Table 2.

**Table 1:** Risk factors associated with the seroprevalance of *Brucella* spp. based on the univariable mixed-effects logistic regression analyses using aggregated data from all animals

Variable	Category	No. tested (n)	% Seroprevalence (95% CI)	Odds ratio (95% CI)	P - value
**Sex**					
	Female	444	12.6 (9.7–16.1)	1 (Ref.)	
	Male	88	4.5 (1.3–11.2)	0.3 (0.1–0.8)	0.020
**Species**					
	Cattle	298	13.8 (10.1–18.2)	1 (Ref.)	
	Sheep	118	2.5 (0.5–7.2)	0.1 (0.0–0.5)	0.002
	Goats	106	13.2 (7.4–21.2)	0.7 (0.3–1.7)	0.443
	Camel	10	20.0 (2.5–55.6)	1.3 (0.2–9.6).	0.805
**Age**					
	Adult	419	13.4 (10.3–17.0)	1 (Ref.)	
	Weaner	60	6.7 (1.8–16.2)	0.6 (0.2–1.7)	0.152
	Young	53	0.0 (0.0–6.7)	0.0	0.974
**Pregnancy status**					
	No	372	9.7 (6.9–13.1)	1 (Ref.)	
	Yes	160	15.0 (9.9–21.5)	1.8 (1.0–3.3)	0.043

*Ref* reference category; *CI* lower and upper limits for 95% confidence interval

**Table 2:** Animal-level seroprevalence of *Brucella* spp. in various livestock species

Variable	Level	Livestock species											
		Cattle			Sheep			Goats			Camels		
		n	% Seroprevalence (95% CI)	***P***-value	n	% Seroprevalence (95% CI)	***P***- value	n	% Seroprevalence (95% CI)	***P***-value	n	% Seroprevalence (95% CI)	***P***-value
**Sex**													
	Male	48	6.3 (1.3–17.2)	0.113	13	7.7 (0.2–36.0-)	0.298	27	0.0 (0.0–12.8)	0.019	0	0.0	1
	Female	250	15.2 (11.0–20.3)		105	1.9 (0.2–6.7)		79	17.7 (10.0–27.9)		10	20.0 (2.5–55.6)	
**Age**													
	Young	25	0.0 (0.0–13.7)	0.031	16	0.0 (0.0–20.6)	0.643	12	0.0 (0.0–26.5)	0.031	0	0.0	1
	Weaners	31	6.5 (0.8–21.4)		18	5.6 (0.1–27.3)		11	9.1 (0.2–41.3)		0	0.0	
	Adults	242	16.1 (11.7–21.4)		84	2.4 (0.3–8.3)		83	15.7 (8.6–25.3)		10	20.0 (2.5–55.6)	
**Pregnancy status**													
	No	212	12.3 (8.1–17.5)	0.267	86	0.0 (0.0–10.9)	0.562	72	8.3 (3.1–17.3)	0.061	2	50 (1.3–98.7)	1
	Yes	86	17.4 (10.1–27.1)		32	3.5 (0.7–9.9)		34	23.5 (10.7–41.2)		8	12.5 (0.3–52.7)	

*n* number of animals tested; *CI* lower and upper limits for 95% confidence intervals

There was a substantial level of agreement between the three serological tests (Cohen’s Kappa statistic k = 0.78). Nevertheless, the proportion of seropositive animals detected by the three tests differed significantly (Cochran’s Q test = 18.5, df = 2, *P* < 0.001). Further post-hoc pair-wise analysis using McNemar’s χ^2^ showed significant differences between RBPT and mRBPT (P < 0.001), RBPT and iELISA (*P* = 0.001), but not between mRBPT and iELISA (*p* = 0.302). Overall, more seropositive animals were detected by the iELISA (10.3%; 95% CI; 7.7–13.0), followed in order by mRBPT (9.4%; 95% CI; 7.1–11.8) and RBPT (7.0%; 95% CI; 5.1–9.1).

A total of 60 seropositive samples were further tested using real-time PCR-based assays. The real-time PCR assay targeting the genus-specific (*bcsp31*) gene detected genus *Brucella* DNA in 49 (81.7%) samples; all of which tested positive for *B. abortus*. There was no *B. melitensis* DNA detected in any of these samples.

### Risk factor analysis

Table 1 shows the results of the independent variables assessed for their association with *Brucella* spp. seroprevalence using univariable mixed-effects logistic regression models. The results of the final multivariable logistic regression model showed that *Brucella* spp. seropositivity was significantly lower among male animals (*P* = 0.012) compared to females (Table 3). Among the livestock species sampled, sheep had statistically significant lower odds of *Brucella* spp. seropositivity than cattle (Table 3). The ICC for within-herd/flock clustering of animals was estimated to be 0.10 (95% CI; 0.02–0.13).

**Table 3:** Results of multivariable mixed-effects logistic regression analysis showing predictors found to be significantly associated with the seroprevalance of *Brucella* spp.

Variable	Category	Odds ratio (95% CI)	***P***- value
**Fixed effects**			
**Sex**			
	Female	1 (Ref.)	
	Male	0.2 (0.1–0.7)	0.012
**Species**			
	Cattle	1 (Ref.)	
	Sheep	0.1 (0.1–0.4)	0.002
	Goats	0.7 (0.3–1.9)	0.502
	Camel	1.1 (0.1–8.6)	0.945

*Ref* reference category; *CI* lower and upper limits for 95% confidence intervals

The estimated variance for the random effect variable (household ID) was 0.38 (SE = 0.03)

### *Brucella* infection incidence results

Table 4 shows the number of observed *Brucella* infection cases and the estimated animal-months at risk stratified by livestock species, sex and age. The estimated overall incidence rate of *Brucella* infection in all animals was 0.024 (95% CI; 0.014–0.037) cases per animal-month at risk. The incidence of *Brucella* infection in camel, cattle, goats and sheep were 0.053 (95% CI; 0.022–0.104), 0.028 (95% CI; 0.010–0.061), 0.013 (95% CI; 0.003–0.036) and 0.006 (95% CI; 0.0002–0.034) cases per animal-month at risk, respectively (Table 4). Considering animal sex, the incidence rate of *Brucella* infection were respectively 0.020 (95% CI; 0.009–0.036) and 0.016 (95% CI; 0.004–0.091) among females and males, while based on age, young animals had a slightly higher incidence rate compared to adults (Table 4).

**Table 4:** The number of animals by sex, species and age recruited for the longitudinal study and their respective estimates of animal time at risk (months), number of observed cases and *Brucella* infection incidence rate

Variable	Levels	n	Animal-time	Cases	Incidence rate	
					Estimate	95% Confidence interval
**Sex**	Male	8	61.60	1	0.016	0.0004–0.091
	Female	71	549.40	11	0.020	0.009–0.036
**Species**	Cattle	31	214.03	6	0.028	0.010–0.061
	Camels	30	151.00	8	0.053	0.022–0.104
	Goats	32	237.40	3	0.013	0.003–0.036
	Sheep	22	161.00	1	0.006	0.0002–0.034
**Age**	Young	11	74.30	2	0.026	0.003–0.097
	Adult	62	536.7	10	0.019	0.009–0.034

The results of the univariable Cox regression analysis are shown in supplementary file 1. Among the investigated risk factors, only species (camels and cattle) was identified as a significant predictor of *Brucella* spp. exposure in animals by multivariable Cox regression analysis (Table 5). The results of the global test used to assess the proportional hazard assumption indicated that this assumption was satisfied (χ^2^ = 7.4, df = 5, *P* = 0.190). Also, all the investigated covariates had *p*-values of > 0.05.

**Table 5:** Outputs of a final multivariable model fitted to the longitudinal data illustrating adjusted hazard rate ratios of *Brucella* spp. exposure in recruited animals

Variable	Levels	Hazard Rate Ratio		***P*** - value
		Estimate	95% Confidence interval	
**Sex**	Male	0.55	0.06–4.55	0.58
	Female	1.00	–	
**Species**	Cattle	4.38	1.00–19.11	0.05
	Camels	5.66	1.62–19.78	0.01
	Sheep	0.78	0.09–6.54	0.82
	Goats	1.00	–	
**Age**	Young	2.41	0.35–16.80	0.37
	Adult	1.00	–	

Number of observations 79, number of events 12

The estimated frailty variance for the random effect term (household ID) using maximum likelihood was 0.54 (SE = 0.08)

## Discussion

To the best of our knowledge, this is the first study to determine the incidence of *Brucella* infection in livestock in a pastoral area in Kenya. In both cross-sectional and longitudinal studies, the seropositivity of *Brucella* spp. in animals was due to natural exposure as vaccination of animals against brucellosis is not done in the area, except in a few commercial farms in other parts of the country. The proportion of *Brucella* spp. seropositive animals detected by mRBPT (9.4%) and iELISA (10.3%) did not differ significantly. Both tests also detected a significantly higher number of seropositive animals than RBPT (7.0%). This finding confirms that mRBPT provides comparable results as iELISA, which is known to have higher sensitivity and specificity, and therefore this test (mRBPT) can be used for more surveillance activities in pastoral areas.

The overall animal-level seroprevalence and incidence rate of *Brucella* infection found in this study were 11.3% (95% CI; 8.6–14.0) and 0.024 (95% CI; 0.014–0.037) cases per animal-month at risk, respectively. While estimates of *Brucella* infection incidence in livestock remain largely unknown in many developing countries including Kenya, partly due to weak surveillance systems and under-reporting, the overall seroprevalence of *Brucella* spp. found in this study was within the ranges previously reported in other pastoral areas (e.g., 7.5 to 40%) in Africa [8]. Both findings confirm that brucellosis is prevalent in the area. Furthermore, animal infections were also clustered within herds/flocks (ICC = 0.10), in agreement with other studies [14, 19]. Infections of animals by *Brucella* spp. could cause high livestock production losses since this disease is contagious and many animals within a herd could become infected [2]. For example, it is estimated that about 20% of cattle in herds with high exposure levels (> 30%) could abort, while milk yields could reduce by 20–25% among aborting animals [5]. Livestock infections by *Brucella* spp. also poses a continuous risk for humans [20], but this study could not confirm this linkage because there was no human component. However, earlier studies conducted in resource-limited areas have found livestock infections to be positively correlated with humans’ exposure [13, 21]. Human infections could occur through direct contact with sick animals or their fluids while relieving dystocia, disposing aborted material, but also by eating undercooked meat, raw/contaminated milk, or dairy products [22]. Indeed, a previous study conducted in the area (Isiolo and Marsabit Counties) detected *B. abortus* and *B. melitensis* in milk samples from camels, cattle, sheep and goats [23]. In addition, human brucellosis has also been reported in Isiolo county, among veterinarians, laboratory personnel, and individuals with febrile illness [24].

This study found a significantly higher incidence of *Brucella* infection in camels and cattle compared to sheep and goats. *Brucella* spp. seroprevalence by livestock species also followed a similar pattern as that of incidence. If seroprevalence results were to be interpreted without the incidence data, it could have been concluded that cattle and camels have higher seroprevalences than sheep and goats because they live longer and hence are more likely to manifest cumulative exposures over time. However, the observed similarities in the patterns of *Brucella* seroprevalence and incidence suggests that camels and cattle have a higher force of infection which manifests as a significantly higher incidence. These infection patterns could be attributed to relative susceptibility of the livestock species sampled in the area to the prevalent *Brucella* species reported in our study. Indeed, a recent study implemented in other pastoral areas of Kenya found that cattle and camels were readily infected with *B. abortus* compared to goats and sheep [11]. This observation could also be connected to the livestock grazing lifestyles used by farmers in the area. For example, it was observed during sampling that cattle and camels were normally raised together in pastoral systems unlike sheep and goats which were grazed within farms. Given these production systems, the effective contact rates between susceptible and *Brucella*-infected animals were therefore likely to be higher among cattle and camel herds compared with sheep and goats. This is due to sharing of pasture and watering sources between several herds and/or the uncontrolled movement of livestock that are typical of pastoral production systems [12].

Our real-time PCR results showed that *B. abortus* which primarily infects cattle and camels was more prevalent in the area compared to *B. melitensis* which naturally infects sheep and goats. A total of 11 (18.3%) samples that were positive by serological tests did not amplify with genus-specific primers for *Brucella* species, and also with species-specific primers for both *B. abortus* and *B. melitensis.* This finding could be due to low yields of *Brucella* DNA in serum samples [25]. The World Organisation for Animal Health (OIE) recommends the use of sequential ELISA tests, as employed in this study, to confirm exposure of animals to *Brucella*. PCR test, though conclusive compared to ELISA, may also not be sensitive enough to pick some of the infections that could become sequestered in tissues [26]. Nonetheless, the detection of *B. abortus* DNA in sheep and goats indicated cross-species transmission (spill-over) from cattle or camels to these hosts which is commonly reported in mixed livestock production systems [11, 27, 28]. However, more studies need to be conducted to determine the relative transmission rates of the various *Brucella* spp. between multiple host species. The higher prevalence of *B. abortus* in livestock in the area compared to other *Brucella* spp. also suggest the presence of underlying biological or ecological mechanisms that influences *Brucella* infection. For example, *B. abortus* pathogen has also been shown to survive in the environment (soil, vegetation) for a long period of time (e.g., 21–81 days) depending on soil moisture, temperature and sunlight [29]. The environmental persistence of *B. abortus* could also indirectly increase the transmission levels of this pathogen if contaminated pastures or watering sources are shared between animals. This would be one entry point for studies on *Brucella* ecology to be conducted to generate more knowledge on how environment influences *Brucella* epidemiology.

The results from the cross-sectional survey showed that *Brucella* spp. seropositivity was significantly associated with animals’ age and sex; adults and female animals had higher levels of exposure compared to young animals and males, respectively. Older animals probably had longer exposure time compared with young ones which could have increased their chances of infection, while females could have had repeated exposure to *Brucella* spp. as they are more likely to stay longer in a herd than males since they are kept for breeding purposes [13]. Besides, the results obtained from multivariable Cox regression analysis did not show significant associations between *Brucella* spp. exposure and animals’ sex or age given that the procedure used to estimate incidence through the calibration of animal-time at risk provided a reliable comparison of the risk of exposure between animals.

The main limitation of this study is that the seroprevalences of *Brucella* spp. in sheep, goats, and camels were estimated using smaller sample sizes than required. This could have led to a low statistical power [30]. Also, a fairly large number of animals were sampled per herd in a few herds which could also have lowered the precision of seroprevalence estimate. Furthermore, the use of a parallel approach to determine the seropositivity of *Brucella* spp. in animals, and to select seronegative animals for follow-up in the longitudinal phase of the study, could also have led to the overestimation of seroprevalence due to low specificity.

## Conclusion

The *Brucella* infection incidence and seroprevalance estimates obtained in this study demonstrated that brucellosis is prevalent in the area. *Brucella* infection incidence was significantly higher among camels and cattle compared to sheep and goats. *Brucella abortus* was more prevalent than *B. melitensis*. Given that livestock infections by *Brucella* spp*.* poses a public health risk for the livestock keepers in Isiolo County, further One Health surveillance studies are required to determine exposure and incidence of this pathogen in humans and to inform control interventions. Vaccination of livestock which is rarely implemented in the area is also recommended because infected livestock animals are the key sources of infections in humans.

## Methods

### Study area

**Fig. 1:**
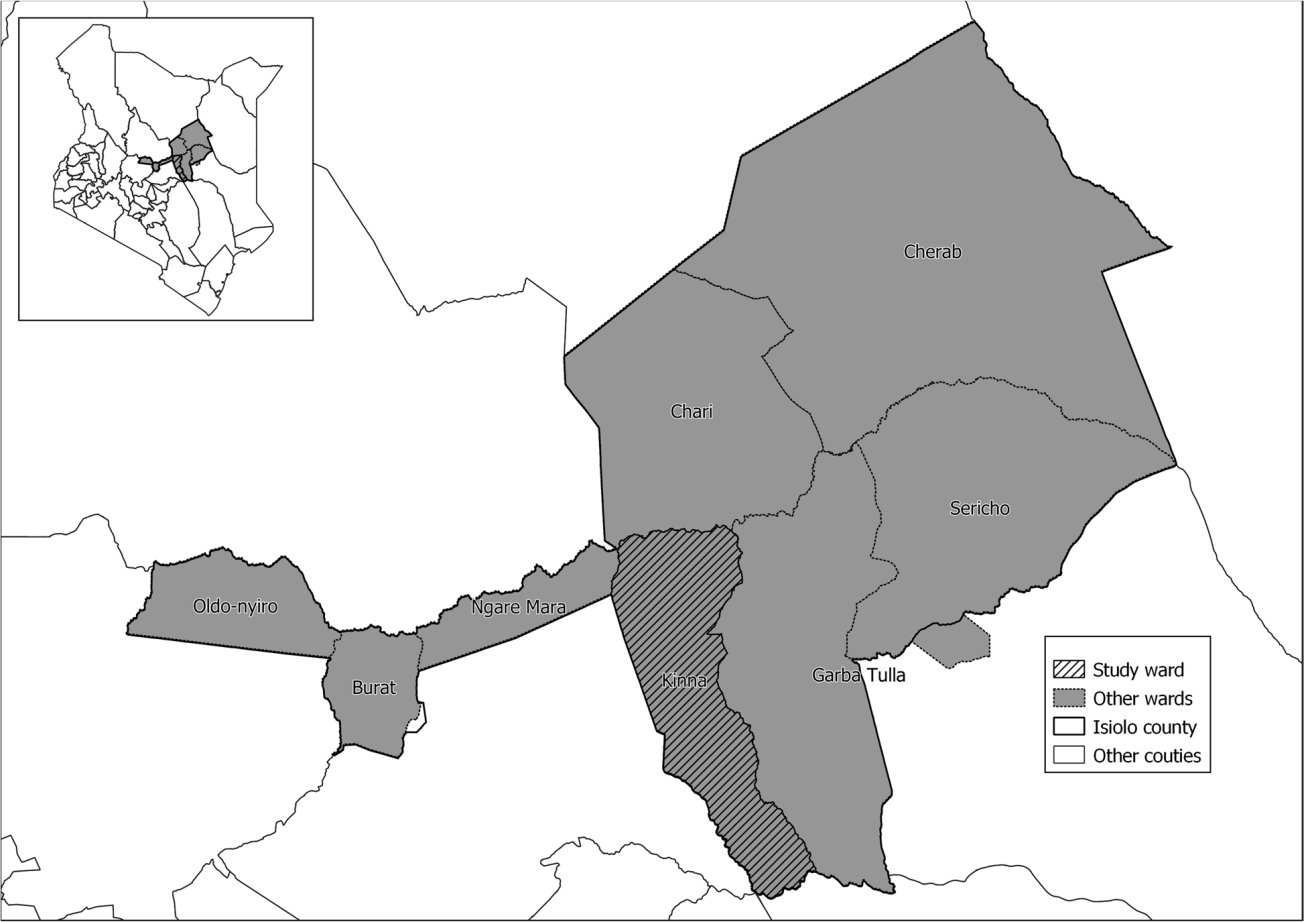
Map showing the location of Kinna ward in Isiolo County

This study was conducted in Kinna ward in Isiolo County, northern Kenya (Fig. 1). The area was selected purposively due to good accessibility and reliable security. In addition, a previous survey that involved the screening of milk for *Brucella* spp. using milk ring test and real time PCR indicated that Kinna had a higher prevalence of *Brucella* spp. compared to other areas that were surveyed in Isiolo and the neighbouring Marsabit counties [23]. Pastoral livestock production system is the main cultural and economic activity for the local people because the area is semi-arid [31]. The average annual rainfall is 580 mm [31], and ranges between 350 and 600 mm [32]. Rainfall in the area has a bimodal distribution; long rains occur from March to May while the short rains occur in November to December [32]. The mean annual temperature in the county range between 24 °C and 30 °C [33].

### Study design and sampling procedure

This study used both cross sectional and longitudinal study designs. The cross-sectional survey was done in December 2017 as a preliminary step to select animals for the longitudinal phase of the study which was conducted between December 2017 and December 2018. The first longitudinal follow-up of negative animals was initiated immediately after the cross-sectional study. The sample size required for the cross-sectional survey was estimated using the formula; n = (1.96)^2^p(1-p)/d^2^ [34]. Based on previous seroprevalence surveys, the expected seroprevalences (p) of *Brucella* spp. in camels, cattle, sheep and goats were 10.3, 16.9, 16.1 and 11.9% [3], while the precision (d) of the test was set at 0.05. The initial sample sizes estimated for each livestock species were adjusted for design effects (DE) to account for the within-household clustering of observations. We derived the DE using the formula DE = 1 + ρ(m-1)), where ρ is the estimated intra-cluster correlation coefficient (ICC) at the household level and assumed that three animals (m) would be sampled in each cluster (household) [35]. An ICC of 0.1 was used to estimate the design effect; this value was adopted from previous similar studies [36, 37]. In general, the ICC values for infectious diseases range from 0.05 to 0.2, except for highly infectious pathogens that could exceed 0.5 [38]. A sample size of 871 animals, including 170 camels, 259 cattle, 249 sheep and 193 goats, was required after adjustment of the initial sample sizes for design effect. However, given that this study used archived sera from a previous study, all the 841 sera samples comprising 382 cattle, 100 camels, 185 sheep and 174 goats that had been collected from a few households (herds) in that study, were all included. Households with at least cattle, sheep and goats were included in the sampling frame since these are the common livestock species found in the area. All the animals sampled during the cross-sectional survey were ear-tagged for easy identification.

For the longitudinal study, animals from each species that were seronegative for *Brucella* spp. by all the three serological tests, from the samples used in the cross-sectional survey, were randomly selected. In total, these included 31 cattle, 22 sheep, 32 goats and 30 camels. These animals were sampled at monthly intervals for a period of 1 year.

### Sample collection

In both cross-sectional and longitudinal studies, about 10 ml venous blood samples were collected from all the animals recruited in plain vacutainers through jugular venepuncture. For the longitudinal study, sampling was done at monthly intervals. In each event, data on animals’ sex, age (young, weaner or adult), pregnancy status (yes/no) and species of the animals kept in the source herd were also obtained using a questionnaire. Serum was extracted from the blood samples after centrifugation at 5000 rpm for 10 min. The samples were transported at − 20 °C to the International Livestock Research Institute (ILRI), Nairobi for laboratory analysis.

### Serological testing

Serum samples collected in the cross-sectional survey were tested for antibodies against *Brucella* spp. using three serological tests – conventional RBPT, modified RBPT and indirect ELISA (iELISA). Those collected in the longitudinal study were tested using iELISA only.

The conventional RBPT followed the procedure described by Nielsen [39]. In brief, 25 μl of the serum sample and an equal volume of the Rose Bengal reagent (antigen) (IDvet Innovative Diagnostics, France) were dispensed onto a white tile next to each other using micropipettes and sterile disposable tips. A sterile applicator stick was then used to mix the test serum sample and the reagent, followed by gentle agitation of the tile for 4 min. Samples showing any visible agglutination to the antigen within the 4 min were classified as positive while those with no agglutination were classified as negative. Serum samples were also re-tested using a modified RBPT (mRBPT) [40]. The testing procedure used for the mRBPT was the same as that of the conventional RBPT described above, except that 75 μl of the serum sample was mixed with 25 μl of the Rose Bengal reagent in each test.

The iELISA technique tested samples for anti-*Brucella* spp. antibodies (IgG1); this used multispecies IDvet kit (IDvet Innovative Diagnostics, France) which could detect infections with either *B. abortus, B. melitensis* or *B. suis.* In brief, we analysed the test and reference sera (positive and negative controls) in duplicates for each test plate and measured the optical densities (ODs) of all the wells at 450 nm. The ratio of the OD of test serum (S) to that of positive control (P) expressed as a percentage was calculated using the formula below:

1 $$Percentage\,{S}\left/{P}\right.=\kern0.5em \left(\frac{\mathrm{mean}\kern0.5em {\mathrm{OD}}_{450}\kern0.5em \mathrm{of}\kern0.5em \mathrm{test}\kern0.5em \mathrm{sample}\kern0.5em \hbox{-} \kern0.5em \mathrm{mean}\kern0.5em {\mathrm{OD}}_{450}\kern0.5em \mathrm{of}\kern0.5em \mathrm{negative}\kern0.5em \mathrm{control}}{\mathrm{mean}\kern0.5em {\mathrm{OD}}_{450}\kern0.5em \mathrm{of}\kern0.5em \mathrm{positive}\kern0.5em \mathrm{control}\kern0.5em \hbox{-} \kern0.5em \mathrm{mean}\kern0.5em {\mathrm{OD}}_{450}\kern0.5em \mathrm{of}\kern0.5em \mathrm{negative}\kern0.5em \mathrm{control}}\right)\kern0.5em \times 100$$

In the case of the cross-sectional survey, we classified animals as negative if ${S}\left/{P}\right.$ was ≤110%, inconclusive (borderline) if between 110 and 120% and positive if ≥120% as recommended by the manufacturer. We re-tested animals with inconclusive iELISA results; those that returned borderline results after re-testing were included as negatives in the analysis. For the longitudinal survey, a new infection (seroconversion) among recruited animals was determined by a positive iELISA test following a negative iELISA result in the previous sampling period.

The diagnostic sensitivity and specificity estimates of these tests were, 87%/97.8%, 98.9%/100%, 92%/100% and 97.8%/100% in camels, cattle, sheep and goats, respectively, for conventional RBPT and 97.2%/99.8%, 96.6%/100%, 100%/100% and 100%/100% in camels, cattle, sheep and goats, respectively, for iELISA [18, 41]. For mRBPT, there is very limited data on the diagnostic sensitivity and specificity of this test among livestock species.

### Molecular detection of *Brucella* DNA using real-time PCR

Samples that tested positive by any of the above three serological tests were further subjected to real-time PCR-based assays to detect genus *Brucella* DNA and for species identification. Genomic DNA was extracted from these samples using the QIAamp blood DNA extraction kit (Qiagen, USA), following the manufacturer’s instructions. Briefly, 200 μl of each serum sample was mixed with 20 μl proteinase K and 200 μl of lysis buffer. The lysate was then taken through the stages of digestion, deactivation, and elusion, according to the manufacturer’s guidelines. The quality and quantity of the extracted DNA was first determined using Nano-Drop spectrophotometer (ThermoFisher Scientific, USA) before DNA samples were stored at − 20 °C until they could be tested.

We performed real-time PCR on all the extracted DNA samples using an ABI 7500 thermocycler machine (Applied Biosystems, Life Technologies, Singapore). The sequences of the oligonucleotide primers and probes used in this study are presented in Table 6. The DNA samples were first amplified using genus-specific primers that targeted the *bcsp31* gene to detect *Brucella* DNA at the genus-level. All the DNA samples that tested positive for genus *Brucella* were further amplified using species-specific primers that targeted the *alkB* and *BMEI1162* genes for *B. abortus*, and *B. melitensis,* respectively [42] (Table 6). The PCR reactions for both the genus and species-specific assays were performed in duplicate, using 20 μl reaction volume containing; 10 μl of 2X PerfeCTa qPCR masterMix, 0.5 μl of each of the pairs of primers (10 nM), 0.25 μl of each of the three probes (10 nM), 2.25 μl of nuclease free water and 4 μl of the (extracted) DNA template. The amplification conditions were as follows; one cycle at 95 °C for 10 min as initial denaturation followed by 40 cycles at 95 °C for 15 s for denaturation, and 1 min for both annealing and extension at 60 °C. Reference strains of *B. abortus* 544 and *B. melitensis* 16 M (from Friedrich-Loeffler-Institute, Germany) were included in all the PCR runs as positive controls, alongside the non-template negative controls. Samples that showed clear amplification plot, accompanied with a cycle threshold (ct) value of < 39 were considered as positive.

**Table 6:** Sequences of oligonucleotide primers and probes used in real-time PCR

Target	Gene target	Forward primer	Reverse primer	Probe	Labels	Reference
*Genus* *Brucella*	*bcsp31* gene	5’GCTCGGTTGCCAATATCAATGC3’	5’GGGTAAAGCGTCGCCAGAAG3’	5’AAATCTTCCACCTTGCCCTTGCCATCA3’	5’Fluorophore-6-FAM, 3’Quencher BHQ1	[42]
*B. abortus*	*alkB* gene	5’GCGGCTTTTCTATCACGGTATTC3’	5’CATGCGCTATGATCTGGTTACG3’	5’CGCTCATGCTCGCCAGACTTCAATG3’	5’HEX 3’BHQ1	[42]
*B. melitensis*	BMEI1162 gene	5’AACAAGCGGCACCCCTAAAA3’	5’CATGCGCTATGATCTGGTTACG3’	5’CAGGAGTGTTTCGGCTCAGAATAATCCACA3’	5’Texas Red 3’BHQ2	[42]

### Statistical analysis

Data entered into Microsoft Excel 2016 was first cleaned before being imported into R statistical software, version 3.6.0 [43] for analysis. All descriptive analyses including the calculation of apparent seroprevalence of *Brucella* spp. was done using the *CrossTable* function in *gmodels* package [44], while the 95% confidence intervals (CI) of the respective estimates were adjusted for within-household clustering using the *epi.conf* function in *epiR* package [45]. The outcome variable (apparent animal-level seroprevalence of *Brucella* spp.) used in our analysis was based on paralleled interpreted results of both conventional RBPT and iELISA. Animals (camels, cattle, sheep and goats) were classified as positive if they had anti-*Brucella* spp. antibodies by either RBPT, or iELISA tests, and negative if no anti-*Brucella* spp. antibodies were detected by both tests. We calculated the true seroprevalance (TP) of *Brucella* spp. for each livestock species from the apparent animal-level seroprevalence (AP) using the formula below;

$$\mathrm{TP}=\kern0.5em \frac{\mathrm{AP}\hbox{-} \left(1\hbox{-} \mathrm{Sp}1\right)\kern0.5em \left(1\hbox{-} \mathrm{Sp}2\right)}{\mathrm{Se}1\kern0.5em \times \kern0.5em \mathrm{Se}2\kern0.5em \hbox{-} \kern0.5em \left(1\hbox{-} \mathrm{Sp}\mathrm{c}1\right)\kern0.5em \left(1\hbox{-} \mathrm{Sp}2\right)}$$

where AP is the apparent seroprevalence; Se1 and Sp1 denoted sensitivity and specificity estimates of the conventional RBPT, respectively, while Se2 and Sp2 were the respective sensitivity and specificity estimates of the iELISA test for each livestock species [46]. The level of agreement between the three serological tests was estimated using Cohen’s Kappa statistic. We also used the Cochran’s Q test to compare the proportion of *Brucella* spp. seropositive animals detected by the three tests followed by post-hoc pairwise comparisons of the tests using McNemar’s χ^2^. Fisher’s exact test was also used to determine the association between categorical predictors (animal’s sex, age, pregnancy status, and species) and the outcome variable. The aggregated data from all the animals was also further subset by the livestock species and the above categorical predictors assessed for their association with the outcome variable.

For the cross sectional data, risk factor analysis was done at the animal-level. We did not perform analysis at herd-level. The categorical variables listed above were first tested for their independent association with *Brucella* spp. seropositivity using univariable mixed-effects logistic regression models. Variables with *p*-value ≤0.15 in the univariable models [34], were further analysed using a multivariable mixed-effects logistic regression model. Data were fitted in both univariable and multivariable models using the *glmer* function in the *lme4* package [47], with the household ID (representing herds/flocks) being entered as a random variable (random effect) to account for the within-herd/flock clustering of observations. The final multivariable model selected comprised only significant covariates (*p* ≤ 0.05) and was used to estimate the intra-cluster correlation coefficient (ICC) due to the clustering of animals within herds/flocks. The variance components of this model were extracted using the *re_var* function in the *lme4* package [47], and the ICC estimated through bootstrap simulation.

For the analysis of the longitudinal data, we first removed cases that were classified as being positive during the cross-sectional study to remain with uninfected animals. Animals were followed on monthly basis until they got exposed. If seroconversion took place between two sampling dates, we used the second sampling date as the time at which the animal was exposed to *Brucella* spp. Animal-time (in months) at risk for each animal was obtained and aggregated to obtain an exact denominator for the overall *Brucella* infection incidence. The numerator was the total number of new *Brucella* spp. cases recorded during the follow-up period. The estimation of the incidence rates with their respective 95% CI (overall incidence as well as by livestock species, sex and age) were performed using the *epi.conf* function in *epiR* package [45].

This study also determined the hazard rate ratio for the above categorical variables using univariable and multivariable Cox proportional hazards models. In these analyses, *Brucella* spp. exposure in animals and time at which the exposure was detected, were both included in the Cox regression models as the outcome of interest. We fitted data to these models using the *coxph* function in the *survival* package [48] and accounted for within-herd/flock clustering of animals using the household ID as a random effect. The proportional hazard assumption was evaluated statistically for each covariate and globally using the *cox.zph* function in *survival* package [48].

## Supplementary Information

**Additional file 1.**

## Data Availability

The datasets used and/or analysed during the current study are available from the corresponding author on reasonable request.

## Abbreviations

RBPT: Rose Bengal Plate Test
mRBPT: Modified Rose Bengal Plate Test
iELISA: Indirect Enzyme Linked Immunosorbent Assay
ICC: Intra-cluster correlation coefficient
ILRI: International Livestock Research Institute
PCR: Polymerase Chain Reaction
