# Correction: Incidence of *Brucella* infection in various livestock species raised under the pastoral production system in Isiolo County, Kenya

**DOI:** 10.1186/s12917-022-03228-1

**Published:** 2022-05-20

**Authors:** Josiah Njeru, Daniel Nthiwa, James Akoko, Harry Oyas, Bernard Bett

**Affiliations:** 1 International Livestock Research Institute, Nairobi, Kenya; 2 Department of Biological Sciences, University of Embu, Embu, Kenya; 3 Department of Biomedical Sciences and Technology, Maseno University, Kisumu, Kenya; 4 Veterinary Epidemiology and Economics Unit, Directorate of Veterinary Services, Ministry of Agriculture, Livestock and Fisheries, Nairobi, Kenya

**Correction: BMC Vet Res 17, 342 (2021)**

**https://doi.org/10.1186/s12917-021-03036-z**

Following the publication of the original article [1], the authors pointed out several corrections to be made. The lines and paragraphs mentioned below are in reference to the published article.

1. Under abstract, results section (line 3) – please change the word “month” to “months” so that it reads “cases per animal-months at risk”. We have highlighted this word in yellow in the attached PDF file of the article.
2. Under the background section in paragraph 1, kindly delete the statement “through spill-over” in line 22. The statement is stricken through in the attached PDF file of the article.
  **Results. Under descriptive analysis**
3. Paragraph 1, lines 24 to 25 in the statement “Fisher’s exact 2-tailed *p* = 0.002”. Please replace the uppercase P with lowercase p so this appears as follows: ‘‘Fisher’s exact 2-tailed p = 0.002’’.
4. Also, in line 25 in the statement: sex (*P* = 0.027). Please replace the uppercase P with lowercase p so this appears as follows: p = 0.027. The other parts should remain as they are.
5. Paragraph 1, line 27. Please add a semicolon after the CI so that the text appears as follows: (4.5% 95% CI; 1.1–8.4). We have also highlighted this section in yellow in the attached PDF file of the article for easy location and action.
6. Under descriptive results, paragraph 1 lines 31 - 33. Please delete the statement “factors associated with the seroprevalance of Brucella spp. based on the univariable mixed)”. In line 31, put a bracket after the figure 17.0. so that it appears as 13.4%; 95% CI; 10.3-17.0). We have stricken thorough this statement in the attached PDF file for easy location and action by deletion.
7. Paragraph 2 under descriptive analysis, line 5. Under the statement: (Cochran’s Q test = 18.5, df = 2, *P* < 0.001). Please replace the uppercase P with lowercase p so this appears as: (Cochran’s Q test = 18.5, df = 2, p < 0.001). In lines 8 to 9 in the same paragraph under the statement: mRBPT (P < 0.001), RBPT and iELISA (*P* = 0.001), please replace uppercase P with lowercase p so that this appears as follows: mRBPT (p < 0.001), RBPT and iELISA (p = 0.001).
8. **Under the heading: Risk factor analysis, line 7.** Please replace uppercase P with lowercase p in the figures P = 0.012 so that this appears as p = 0.012. This section has been highlighted in the attached PDF file for easy identification and action.
9. **Under the heading:** *Brucella* infection incidence results, paragraph 1, line 5. Change the word “month'' to “months'' so that this statement reads: “animal-months at risk''. In line 9, please also change the word “month'' to “months''. Paragraph 2, line 8, under the figures (χ2 = 7.4, df = 5, *P* = 0.190). Kindly replace uppercase P with lowercase p so that this reads (χ2 = 7.4, df = 5, *p* = 0.190).
10. Under discussion section, paragraph 2, line 4. Replace the word “month'' with “months''.
11. Under statistical analysis section, page 9. In the denominator part of the below formula, please delete the c in Spc1 so that this becomes Sp1. The other parts of the formula are correct. In the attached PDF file, we have deleted letter c in the formula.

$$\mathrm{TP}=\frac{\mathrm{AP}\hbox{-} \left(1\hbox{-} \mathrm{Sp}1\right)\;\left(1\hbox{-} \mathrm{Sp}2\right)}{\mathrm{Se}1\times \mathrm{Se}2\hbox{-} \left(1\hbox{-} \mathrm{Sp}1\right)\;\left(1\hbox{-} \mathrm{Sp}2\right)}$$

The original article has been corrected.
