# Supplementary material for: Incidence of Brucella infection in various livestock species raised under the pastoral production system in Isiolo County, Kenya
Source: BMC Vet Res. 2021 Oct 30;17:342. doi: 10.1186/s12917-021-03036-z (PMC8556948; doi:10.1186/s12917-021-03036-z)
Supplement: Supplementary file 1 — Additional file 1. [file 12917_2021_3036_MOESM1_ESM.docx]

**Supplementary file 1**

**Crude hazard rate ratios obtained from univariable analyses conducted using the Cox proportional hazards model**

| **Variable** | **Levels** | **Hazard Rate Ratio** | | **P - value** |
| --- | --- | --- | --- | --- |
|  |  | **Estimate** | **95% Confidence interval** |  |
| **Sex** | Male | 0.74 | 0.095 – 5.74 | 0.72 |
|  | Female | 1.00 | - |  |
|  |  |  |  |  |
| **Species** | Cattle | 2.36 | 0.67 – 8.26 | 0.18 |
|  | Camels | 4.95 | 1.70 – 14.36 | 0.00 |
|  | Sheep | 0.48 | 0.12 – 1.97 | 0.30 |
|  | Goats | 1.00 | - |  |
|  |  |  |  |  |
| **Age** | Young | 1.35 | 0.29 – 6.16 | 0.70 |
|  | Adult | 1.00 | - |  |
